# Injuries caused by sea urchins on the Brazilian coast: advances in the development of therapeutic methods and prevention of wounds

**DOI:** 10.1590/0037-8682-0314-2025

**Published:** 2026-02-02

**Authors:** Vidal Haddad

**Affiliations:** 1 Universidade Estadual Paulista, Faculdade de Medicina de Botucatu, Departamento de Dermatologia e Infectologia, São Paulo, SP, Brasil.

**Keywords:** Sea urchins, Wounds and injuries, Emergency treatment, Bacterial infections, Trauma prevention

## Abstract

**Background:**

Sea urchins inhabit rocky areas and lagoons near tourist bathing sites. These animals have sharp spicules on their surfaces that cause injuries to bathers.

**Methods:**

Over 24 months, the author identified sea urchin species and mapped the beaches where injuries occurred. Samples of spicules were stored. In some patients, spicules were extracted using fine watchmaker's forceps. The intensity of pain and late infections were also recorded.

**Results:**

Sixty-two patients (48 men, 77.42%) experienced injuries. The plantar regions were affected in 58 (93.55%) patients. In 26 cases, spicules were collected, being of the species *Echinometra lucunter*, which is not venomous. Twenty patients were randomly selected for spicule extraction using No. 10 watchmaker's forceps. Larger fragments were easily and practically painlessly removed; however, the forceps were not fully effective for small fragments.

**Conclusions:**

Injuries caused by black sea urchins were the most common among those caused by marine animals in bathers. The unique species was *Echinometra lucunter*, which is not venomous. The penetration of spicules occurs mainly in the plantar regions and is a significant traumatic factor, with the possibility of secondary infections and foreign body granulomas. The use of No. 10 (watchmaker's) forceps was equivalent to the needle method but much less painful and traumatic. The creation of leaflets and the placement of posters at beaches with sea urchin colonies could prevent these wounds and should be attempted by the City Councils of coastal cities.

## INTRODUCTION

Reports of trauma and envenomation by aquatic animals worldwide are sporadic and do not have a sequence capable of allowing in-depth studies on the incidence of injuries^1^ ^-^ ^7^. The main animals causing these injuries and their clinical characteristics have only recently been studied, providing new possibilities for the development of effective therapeutic and preventive measures^6^ ^,^ ^7^.

The first series of injuries by marine animals observed in Brazil was recorded in Ubatuba, on the northern coast of the state of São Paulo, Brazil, a municipality with many beaches frequented by bathers (latitude: -23.4339, longitude: -45.0857 23° 26′ 2″ South, 45° 5′ 9″ West). In the early 2000s, approximately 150 patients were treated for injuries caused by sea urchins, cnidarians, and venomous fish^1^ ^,^ ^2^ ^,^ ^3^ ^,^ ^6^. Since then, several new epidemiological and clinical observations have been made in this area; however, the percentages and clinical characteristics recorded in the initial study have been maintained^7^. For every 1,000 patients treated in emergency rooms in coastal cities, one patient is a victim of a marine animal^1^ ^-^ ^7^. The animals associated with injuries are sea urchins (approximately 50%), Portuguese man-of-war and jellyfish (approximately 25%), and catfish and stingrays (approximately 25%)^1^ ^-^ ^7^. This profile of trauma and envenomation occurred in bathers. The profile of injuries in fishermen is not the same^1^ ^,^ ^4^ ^,^ ^7^.

**FIGURE 1:**
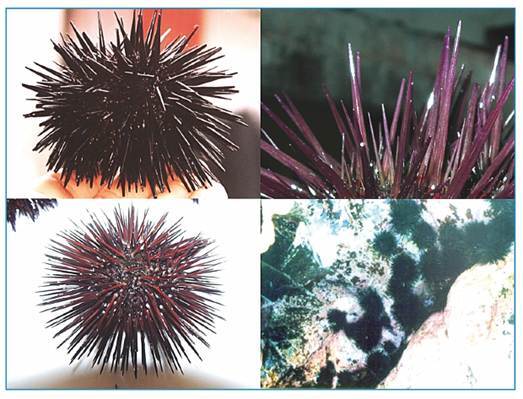
Black sea urchins (*Echinometra lucunter*), detail of body spicules and colony in shallow waters.

Sea urchins are the main cause of wounds among bathers on Brazilian beaches^8^ ^-^ ^10^. Sea urchin species are the same throughout the Brazilian coastline, with black sea urchins (*Echinometra lucunter*) being the most common, accounting for approximately 90% of the animals^8^ ^-^ ^10^. These animals have rounded bodies with numerous black calcium carbonate spicules on their surfaces (Figure 1). These sharp structures can penetrate deeply into the human skin, most commonly in the plantar regions. Bathers step on sea urchins while walking along the rocks that line the beaches as the animals gather in colonies in the small lagoons formed by the tides in these rock formations^8^ ^-^ ^14^.

**FIGURE 2:**
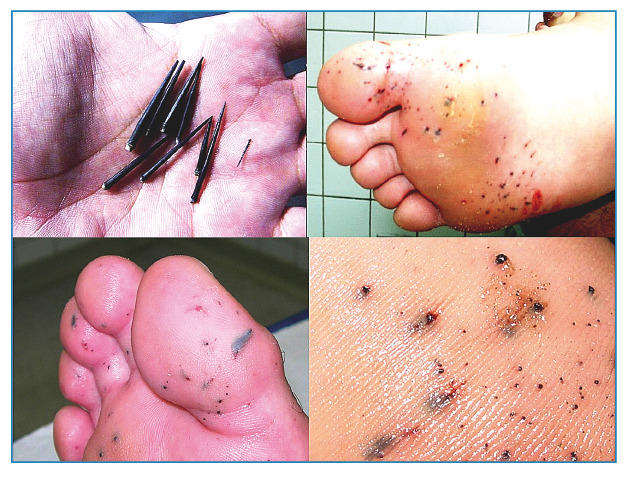
Spicules of black sea urchins and patients with fragments after stepping on sea urchins showing black spots where the spicules penetrated.

Black sea urchins cause traumatic wounds (without envenomation) and are responsible for approximately half of the injuries involving aquatic marine animals treated in emergency rooms of coastal cities (Figure 2)^1^ ^,^ ^8^ ^,^ ^10^ ^,^ ^13^. These injuries are not serious and do not cause systemic repercussions; however, they cause moderate-to-severe local pain when the spicules penetrate and during walking. Late complications, such as foreign body granulomas due to the retention of fragments of the spine (which require surgical interventions for removal and bacterial and fungal infections), may also be observed^1^ ^,^ ^8^ ^,^ ^10^ ^,^ ^13^.

Envenomations by venomous sea urchins cause intense inflammatory processes in the skin, manifesting as erythema, edema, papules, vesicles, and occasional cutaneous necrosis. Cardiotoxicity and neurotoxicity can also occur. They are much rarer in Brazil, as they are associated with species that inhabit the high seas, of the genus *Diadema* ^8^ ^-^ ^10^.

For injuries without envenomation (in almost all cases), it is advisable to remove the spicules immediately^11^. To extract them, superficial scarification is performed at the point of entry of the spicule with a large-caliber hypodermic needle, and the spicules are removed with another needle supported by the first needle, in a process that does not always allow the use of anesthetics and can cause considerable pain^8^ ^-^ ^11^. Some patients have hundreds of spicules in the plantar regions or other regions of the body. Although these injuries are initially treated as emergencies, late infections and fragment retention require outpatient care, as they occur days or weeks after the penetration of spicules ^10^ ^,^ ^11^.

It is necessary to identify the predominant species in the injuries (venomous or not) and map the beaches where these are most frequent in the study region (topography and proximity of bathers to sea urchin colonies on rocks and tidal lagoons). Another important measure is to seek a less aggressive method of extracting the spicules, knowing that the smaller fragments of the spicules are usually expelled by local inflammatory reactions and that several points on the skin are tattoos of black pigments, which penetrate and exit without fragmentation. However, larger spines are associated with bacterial infections and foreign- body granulomas if not removed. Granulomas are a problem and require total excision for complete resolution^8^ ^-^ ^11^.

**FIGURE 3:**
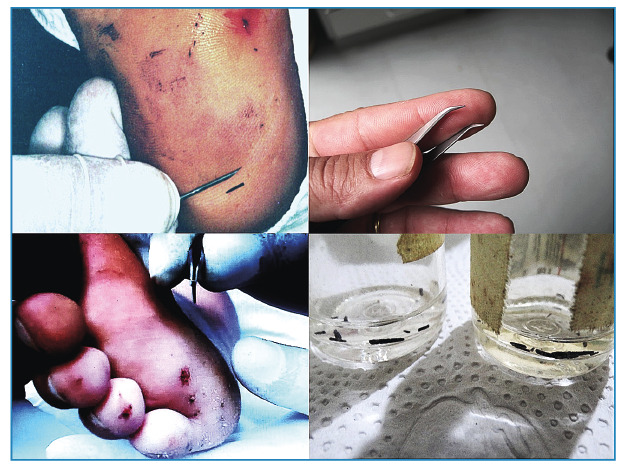
Method of extracting spicules of sea urchins using needles (left) and No. 10 fine forceps (right) and spicules collected for identification.

The primary objective of this study was to prevent and adequately treat sea urchin injuries. To this end, it was necessary to achieve secondary objectives, such as evaluating and mapping the prevalence of sea urchin injuries in swimmers treated at the local Emergency Room in the municipality of Ubatuba, located on the northern coast of the State of São Paulo, categorizing the species of animals, verifying the body topography and severity of the injuries of the victims, and analyzing the therapeutic outcome of removing the spicules with fine surgical forceps with grooves used in hair transplants, called 10 cm watchmaker's forceps (Figure 3).

## METHODS

This study was approved by the Research Ethics Committee of Botucatu School of Medicine (CAAE: 10027819.5.0000.5411, opinion: 3,239,609). During the proposed 24-month period, 64 out of 133 patients injured by marine animals were wounded by sea urchins and sought medical assistance on weekends (when beachgoers visit the beaches in greater numbers). The patients were treated in the Emergency Room of the Santa Casa de Misericórdia Bom Jesus dos Passos in Ubatuba, São Paulo, Brazil.

Demographic data such as sex, age, skin color, origin of the swimmer (local or tourist), beaches where the wounds occurred (detailed mapping of the locations), body topography of the penetration of the spicules, severity of the injuries, and the conduct instituted were recorded. Signs of infection and the intensity of initial pain were also recorded.

The spicules were removed using the specified fine forceps and large-caliber needles and submitted for subsequent identification by a specialist. The effectiveness of spicule removal using fine tweezers was assessed by the presence of remaining spicules on the skin in a new consultation scheduled for seven days after the initial consultation, owing to the uncertain length of stay of tourists in the city.

## RESULTS

During the study period, 133 patients were injured by marine animals, including cnidarians, venomous and poisonous fish, and sea urchins. Of these, 62 individuals (46.61%) were injured by sea urchins, with the majority of trauma occurring in males (48 patients, 77.42%).

Most patients had injuries caused by stepping on colonies of black sea urchins that inhabit lagoons formed by tides between rocks on the coast (60 patients, 93.75%). These colonies are not always visible, especially when the water is murky. Another precipitating factor is falling on colonies of sea urchins when walking in lagoons formed between rocks by tides.

The most affected body regions were the plantar regions in 58 patients (93.55%), with a predominance in the right plantar region (Table 1). The number of spicules varied from less than a dozen to more than a hundred in the most severe case.

**TABLE 1:** Distribution of plantar lesions in the observed patients.

Total plantar lesions	Right plantar region only	Left plantar region only	Both plantar regions
58	31	19	8
93.55%	53.45%	32.75%	13.80%

Four patients with spicules in regions other than the plantar region had lesions on the palms, and one of them had fallen from a rocky cliff onto a colony of sea urchins, with penetration of dozens of spicules in the posterior thoracic region and posterior regions of the arms and legs.

All patients showed black spicule fragments at the puncture points (100%). In 26 cases, the spicules could be collected, being characteristic of the species *Echinometra lucunter*, which is not venomous and the most prevalent along the entire Brazilian coast, because they are closer to the tidal surf area than the other species (Figure 1).

The beaches where trauma occurred were mainly Praia Grande (more than 80% of the patients). This was due to its large size and number of tourists, in addition to the colonies of black sea urchins in the rocky areas that delimit it. Approximately 10% of the 62 accidents occurred at a nearby beach, Praia do Tenório, and the remaining 10% occurred at several beaches, including Praia do Alto, Itamambuca, Perequê-açu, Ilha Anchieta, Saco da Ribeira, and Praia do Félix.

The spicules were extracted using large-caliber needles, using a method commonly used in coastal emergency rooms, with one needle fixing the spicule at the point of penetration and the other used to extract the fragment with upward movement (Figure 3).

The procedure is associated with considerable pain and cannot always extract all spicules, as it is limited to the largest fragments. Local anesthesia is ineffective in these cases, where most lesions are in the plantar regions with multiple foci.

Twenty patients with spicule fragments in the plantar region were randomly selected from the 62 patients in the study to have the spicules extracted using No. 10 watchmaker's forceps (Figure 3).

The larger fragments were easily and practically painlessly removed from these patients; however, this was not the case for the smaller fragments and those with black pigmentation without fragments (tattoos), in which the forceps were not fully effective because of the fragility and breakage of the fragments. Follow-up appointments were scheduled for all patients after one week. However, owing to their short stay during the summer and weekend periods, only 12 patients attended the consultation (19.35% of the total number of sea urchin injuries). Clinical observations are shown in Table 2.

**TABLE 2:** Patients’ evolution after spicule removal.

	Local inflammation discreet	Presence of inflammatory nodules	Presence of secondary infection	Full resolution
Patients with needle withdrawal (6)	4 (66.66%)	0	1 (16.66%)	1 (16.66%)
Patients with removal using No. 10 forceps (6)	5 (83.33%)	0	0	1 (16.66%)

The results obtained in this study are consistent with previously published data. The possibility of secondary infections and foreign-body granulomas is complicated by trauma, despite no envenomations^8^ ^,^ ^10^. There is even more interest in this type of trauma because it is most commonly provoked by marine animals in the country^8^ ^,^ ^10^ ^,^ ^13^ ^,^ ^14^.

Trauma caused by sea urchins does not occur on the sand of the beaches but rather near colonies between rocky shores located between the beaches, which causes a high incidence of spicules in the plantar regions of patients. The right foot is the first to step and thus has a higher incidence of injuries.

The data showed the prevalence of trauma caused by sea urchins, their seasonality, demographic profile, and the effective removal of major spicules with fine forceps. The proportion of revaluations of the spicules present was compared with the historical series of locations using Pearson's chi-squared test. Statistical significance was set at p <0.05.

These results may provide new perspectives for treating a common problem on Brazilian beaches, reducing the difficulties posed by spicule extraction using needles and the presence of late complications. Using the No. 10 forceps simplified and easily removed the larger spicules, which is a breakthrough in treatment. Furthermore, although the extraction results were similar in both methods, the second method is much less aggressive and practically painless, which is a significant advance in solving the problem.

## DISCUSSION

Injuries caused by sea urchins in bathers are the most common among those caused by marine animals. The most commonly involved species was the black sea urchin (pindaúna) of *Echinometra lucu*n*ter*, which is not venomous. The penetration of spicules (and their breakage) occurs mainly in the plantar regions and is a significant traumatic factor, with the possibility of secondary infections and the formation of foreign body granulomas. Removal is necessary, and the persistence of large fragments is a problem that should be addressed as soon as possible. The needle removal method is effective but painful and traumatic. The possibility of using the No. 10 (watchmaker's) forceps is real, and the results were equivalent to the needle method but much less painful and traumatic for patients. The creation of leaflets and the placement of posters at beaches with sea urchin colonies could prevent this type of accident and should be attempted by the City Councils of coastal cities.

## Data Availability

Research data is available upon request.
